# Frontline physician burnout during the COVID-19 pandemic: national survey findings

**DOI:** 10.1186/s12913-022-07728-6

**Published:** 2022-03-19

**Authors:** Joy Melnikow, Andrew Padovani, Marykate Miller

**Affiliations:** 1 Center for Healthcare Policy and Research, Department of Family and Community Medicine, University of California, Davis, USA; 2 Sacramento, California USA; 3 Center for Healthcare Policy and Research, University of California, Davis, USA

**Keywords:** COVID-19, Frontline physicians, Physician burnout, Physician wellbeing

## Abstract

**Background:**

Physician burnout and wellbeing are an ongoing concern. Limited research has reported on the impact of the COVID 19 pandemic on burnout over time among U.S. physicians.

**Methods:**

We surveyed U.S. frontline physicians at two time points (wave one in May–June 2020 and wave two in Dec 2020-Jan 2021) using a validated burnout measure. The survey was emailed to a national stratified random sample of family physicians, internists, hospitalists, intensivists, emergency medicine physicians, and infectious disease physicians. Burnout was assessed with the Professional Fulfillment Index Burnout Composite scale (PFI-BC). Responses were weighted to account for sample design and non-response bias. Random effects and quantile regression analyses were used to estimate change in conditional mean and median PFI-BC scores, adjusting for physician, geographic, and pandemic covariates.

**Results:**

In the random effects regression, conditional mean burnout scores increased in the second wave among all respondents (difference 0.15 (CI: 0.24, 0.57)) and among respondents to both waves (balanced panel) (difference 0.21 (CI: − 0.42, 0.84)). Conditional burnout scores increased in wave 2 among all specialties except for Emergency medicine, with the largest increases among Hospitalists, 0.28 points (CI: − 0.19,0.76) among all respondents and 0.36 (CI: − 0.39,1.11) in the balanced panel, and primary care physicians, 0.21 (CI: − 0.23,0.66) among all respondents and 0.31 (CI: − 0.38,1.00) in the balanced panel. The conditional mean PFI-BC score among hospitalists increased from 1.10 (CI: 0.73,1.46) to 1.38 (CI: 1.02,1.74) in wave 2 in all respondents and from 1.49 (CI: 0.69,2.29) to 1.85 (CI: 1.24,2.46) in the balanced panel, near or above the 1.4 threshold indicating burnout. Findings from quantile regression were consistent with those from random effects.

**Conclusions:**

Rates of physician burnout during the first year of the pandemic increased over time among four of five frontline specialties, with greatest increases among hospitalist and primary care respondents. Our findings, while not statistically significant, were consistent with worsening burnout; both the random effects and quantile regressions produced similar point estimates. Impacts of the ongoing pandemic on physician burnout warrant further research.

## Background

Physician burnout has received growing attention in recent years [1]. The COVID-19 pandemic presents an unprecedented healthcare challenge to the world, and international research has indicated that healthcare workers have experienced depression, anxiety, post-traumatic stress symptoms and greater burnout [2–7]. More limited research has reported on the pandemic’s impact on U.S. physician burnout; most surveys have focused on single health systems or single specialties [8–10]. One national survey solicited survey respondents via social media to evaluate the incidence of depressive symptoms, anxiety, and PTSD at a single time point [11]. As the pandemic has continued, there is a need to track the impact on physician burnout over time.

Burnout is conceptualized as a consequence of chronic stress, with chronic feelings of exhaustion, negative attitudes towards work, and decreased professional efficacy [12]. Among physicians, burnout is associated with decreased well-being and worse quality of patient care [13–15]. One theory of burnout, the Job Demands-Resources (JDR) theory, describes two major elements contributing to burnout: 1) high job demands including workload, role ambiguity, role conflict, role stress, stressful events, and work pressure, combined with 2) limited job resources including social support, autonomy, and skill variety. Lack of control over work, lack of regular feedback, and lack of professional development all contribute to worsening burnout [16]. The COVID-19 pandemic has high potential to worsen all these elements with ongoing surges creating increased and unpredictable job demands in a setting where institutional resources for support are stretched thin.

We conducted an online survey at two time points during the pandemic (June–July 2020 and December 2020-Jan 2021) using a non-proprietary, validated measure of burnout [17] in a national stratified sample of U.S. physicians practicing in frontline specialties. We hypothesized that physician burnout would be higher than pre-pandemic measures and that burnout would increase over time as the pandemic continued.

## Methods

### Sample

A stratified random sample of 10,000 US physicians from a comprehensive list (AMA Physician Masterfile) [18] included 4000 primary care physicians (2000 family physicians and 2000 internists), 1000 hospitalists, 2000 critical care physicians (1000 critical care and 1000 pulmonary intensivists), 2000 emergency medicine physicians, and 1000 infectious disease physicians. Hospitalists, intensivists, infectious disease, and emergency medicine physicians were proportionally oversampled to ensure responses represented these frontline specialties. We simplified specialty categories for analysis by combining family physicians with internists into a category called Primary care, and critical care physicians with pulmonary intensivists into a category called Critical care.

### Survey

We distributed a Qualtrics survey via email at two time points, June–July 2020 (wave 1) and December–January 2021 (wave 2). Each wave of the survey was sent three times over a period of 3 weeks to enhance the response rate. The study design was a repeated cross section with clustering, addressed in the analytic approach as described below.

The survey assessed burnout over the previous 2 weeks with the burnout composite of the Professional Fulfillment Index scale (PFI-BC) [17], a validated, open access measure of physician burnout. The PFI-BC scale averages the work exhaustion and interpersonal disengagement scales. Items are rated from “Not at all” (0) to “Extremely” (4). Respondents were defined as experiencing burnout if their composite score exceeded 1.4, previously defined as a threshold for burnout [17]. Additional open response questions inquired about how physicians’ lives had changed with the pandemic, and what would make things better for their patients and in their work.

### Analysis

Our analytic approach was designed to maximize sample representativeness and reduce bias in identification of rates and changes in specialty burnout over time. The random sample of physicians ensured eligibility for the survey was free of selection bias. Weighting was applied to the respondents to achieve representativeness and to reduce non-response bias: sampling design weights adjusted the sample to be representative of the specialties in our AMA Physician Masterfile sampling frame, and non-response weights addressed bias due to physician's self-selecting to respond [19].

We constructed sample design weights as the inverse probability of selection into the sample. The study sample was stratified by physician specialty, thus the probability a given physician appeared in our sample was a function of the physician’s specialty. For example, the probability any given family physician appeared in our sample was about 2.9% (2000 sampled from a population of 69,400), while the probability a given Hospitalist appeared in our survey was 72.7% (1000 sampled from a population of 1376). Therefore, survey design weights were obtained by computing the inverse of the probability of selection for each specialty. To ensure correct weighting of survey respondents the original specialty categories provided by the AMA were applied rather than the combined specialties used for analysis.

Non-response weights were constructed using entropy balancing [20], a nonparametric generalization of the propensity score weighting approach [21]. Entropy balancing constructs unit weights calibrated to match the mean, variance, and skewness of the full sample. Non-response weights were constructed such that the mean, variance, and skewness of the distribution of osteopathic doctors, females, years in practice, and type of practice among respondents matched the full stratified sample. Entropy balancing was performed with the user-written KMATCH module for Stata [22].

Respondent data were analyzed in a weighted regression controlling for potential confounding bias in burnout scores due to geographic variation in COVID-19 onset, rates, and policies. The analysis controlled for county-specific pandemic data such as the first month with a positive count of COVID-19 cases, the number of cases and growth rate of cases in the 2 weeks prior to survey response, and geographic location.

We mapped each physician’s city and state of residence from the AMA Masterfile data to counties, to adjust for local pandemic-related confounding variation in the intensity of the COVID-19 pandemic. Daily COVID-19 case count data between January 22, 2020 and April 21, 2021 was obtained for all US counties from the COVID-19 Data Repository [23] maintained by the Center for Systems Science and Engineering (CSSE) at John Hopkins University. For each respondent’s county, the COVID-19 case count and growth rate from the 2 weeks prior to the date of survey completion were included in the regression analysis as independent variables. The first month with a positive case count as well as the case count in the first month were included as a fixed effect. Each respondent’s state of residence was mapped to census divisions and included as a fixed effect to account for division-specific variation in pandemic intensity and policy responses.

The following empirical equation was estimated to analyze self-reported physician burnout:

$${\mathrm{Y}}_{\mathrm{i}\mathrm{t}}=\upalpha {\mathrm{w}}_{\mathrm{i}}+{\sum}_{\mathbf{j}=\mathbf{1}}^{\mathbf{S}}{\upbeta}_{\mathrm{j}}{\mathrm{s}}_{\mathrm{i}\mathrm{j}}+{\sum}_{\mathrm{j}=1}^{\mathrm{S}}{\upgamma}_{\mathrm{j}}\left({\mathrm{w}}_{\mathrm{i}}{\mathrm{s}}_{\mathrm{i}\mathrm{j}}\right)+{\mathbf{X}}_{\mathbf{it}}^{\prime}\boldsymbol{\upzeta} +{\updelta}_{\mathrm{i}}+{\mathrm{c}}_{\mathrm{i}}+{\upepsilon}_{\mathrm{i}\mathrm{t}},$$

where i denotes individuals and t denotes time point. Y_it_ is the PFI-BC score for individual i at time point t, w_i_ = 1[t = 2] is a dummy variable taking 1 for time point t = 2, ${\mathrm{s}}_{\mathrm{ij}}=1\left[\mathsf{Specialt}{\mathsf{y}}_{\mathrm{i}}=\mathrm{j}\right]$ is a dummy variable taking 1 when individual i has specialty j and 0 otherwise, **X**_**it**_ denotes the vector of county-level pandemic covariates for individual i at wave t, δ_i_ is a census division fixed effect, c_i_ is the individual-specific error component, and ϵ_it_ is the model error component.

The change in burnout in specialty j from wave 1 to wave 2 is estimated by (α + γ_j_). We estimated the empirical equation by weighted random effects regression [24] with standard errors estimated by the cluster-robust estimator. We report results for both the unbalanced and balanced panels. In addition, we explored heterogeneity in the estimate of (α + γ_j_) at different parts of the PFI-BC distribution with a similar equation estimated by conditional quantile regression with standard errors estimated by the Huber/White/Sandwich estimator; c_i_ - the individual error component - is dropped from the conditional quantile regression. All data processing and analysis was performed with Stata/MP 17™.

Responses to two open-ended questions that appeared in both waves of the survey (“what would make the most difference to you in your work during the COVID-19 pandemic?” and “what would make the most difference right now to your patients during the COVID-19 pandemic?”) were evaluated by thematic analysis conducted using an open coding and constant comparison approach [25]. Three coders independently reviewed the text and met weekly to refine definitions and discuss provisional categories. Codes were created both inductively, emergent codes, and deductively, priori codes. After a final codebook was developed and agreed upon, each coder independently reviewed and coded all responses. Coders then met to discuss and reach consensus through triangulation. Based on the emergent codes, themes and subthemes were produced by collapsing individual codes and creating categories.

## Results

Table 1 shows descriptive statistics of responders and non-responders. Diagnostic measures of balance are included in Table 1 for weighted standardized difference in means and weighted ratio of variances. Balance in mean is achieved when the weighted standardized difference in means is 0, and in variance when the weighted ratio of variances is 1; the entropy balancing estimator achieved approximately perfect balance.

**Table 1:** Descriptive statistics of respondents and non-respondents

	Response status			Weighted balance^a^	
	Did not respond	Responded	Total	Mean^b^	Ratio^c^
N (%)	8264 (94.8)	451 (5.2)	8715 (100.0)		
Medical training					
MD	7663 (92.7)	417 (92.5)	8080 (92.7)		
DO	601 (7.3)	34 (7.5)	635 (7.3)	0.000	1.002
Sex					
Male	5523 (66.8)	259 (57.4)	5782 (66.3)		
Female	2741 (33.2)	192 (42.6)	2933 (33.7)	0.000	1.002
Physician specialty					
Critical care medicine	826 (10.0)	54 (12.0)	880 (10.1)		
Emergency medicine	1586 (19.2)	92 (20.4)	1678 (19.3)	0.000	1.002
Family medicine	1688 (20.4)	79 (17.6)	1767 (20.3)	0.000	1.002
Hospitalist	835 (10.1)	54 (12.0)	889 (10.2)	0.000	1.002
Infectious disease	814 (9.8)	56 (12.4)	870 (10.0)	0.000	1.002
Internal medicine	1684 (20.4)	68 (15.1)	1752 (20.1)	0.000	1.002
Pulmonary critical care	831 (10.1)	47 (10.4)	878 (10.1)	0.000	1.002
Years since residency^d^	17.8 (11.1)	17.5 (10.8)	17.8 (11.1)	0.000	1.002
Type of practice					
Office	5997 (72.6)	315 (70.0)	6312 (72.4)		
Hospital staff	2134 (25.8)	128 (28.4)	2262 (26.0)	0.000	1.002
Teaching	133 (1.6)	7 (1.6)	140 (1.6)	0.000	1.002

^a^ Weighted balance is based on diagnostic output produced by the kmatch module

^b^ Mean is the standard difference in means between weighted respondents and weighted non-respondents; standard difference is 0 when perfectly balanced. Standard difference in means is rounded to 3 significant digits

^c^ Ratio represents the ratio of variances of weighted non-respondents to variance of weighted respondents; ratio is 1 when perfectly balanced. Ratio of variances is rounded to 3 significant digits

^d^ Reports mean Years since residency with standard deviation in parentheses

The survey data from all respondents consisted of an unbalanced panel across physician specialties, with 286 responses in wave 1 and 262 responses in wave 2. After removing bounced and otherwise invalid email addresses, our response rates were 3.3% in wave 1 and 3% in wave 2. Of these 548 total responses, 194 came from 97 physicians who responded to both waves of the survey. We constructed a balanced panel of these 97 physician responders to both waves, henceforth referred to as the “balanced panel”.

Table 2 presents the weighted, unadjusted mean and standard deviation of respondent PFI-BC scores by specialty, along with the proportion of respondents with PFI-BC scores of at least 1.4 - the threshold indicating burnout [17]. This summary of PFI-BC scores was not adjusted for regional differences in pandemic conditions. The mean unconditional PFI-BC score was 1.19 in wave 1 and 1.15 in wave 2, with 37.5 and 33.6% of respondents, respectively, indicating burnout. Emergency medicine physicians reported the highest burnout scores in wave 1 with a mean of 1.49 with 51.4% indicating burnout. Critical care physicians reported the next-highest PFI-BC scores in wave 1 with a mean score of 1.31 with 53.1% indicating burnout. In wave 2, the mean score remained unchanged at 1.32, but the proportion indicating burnout decreased to 42.8%. In both waves, primary care and infectious disease physicians reported the lowest unconditional PFI-BC scores and the smallest proportion of respondents indicating burnout. Burnout decreased most among emergency medicine physicians from 1.49 in wave 1 to 1.29, while burnout increased the most among Hospitalists. Mean PFI-BC among hospitalists rose from 1.23 in wave 1 to 1.37 in wave 2, and the proportion of respondents with scores indicative of burnout increased from 28.2% in wave 1 to 41.5% in wave 2.

**Table 2:** Summary statistics for PFI Burnout Composite score and proportion of respondents reporting burnout by physician specialty

	Survey Wave		
	Wave 1	Wave 2	Total
Mean (SD) PFI-BC score			
Emergency medicine	1.49 (0.87)	1.29 (0.85)	1.38 (0.86)
Critical care^a^	1.31 (0.74)	1.32 (0.83)	1.32 (0.78)
Primary care^b^	1.13 (0.90)	1.10 (0.92)	1.11 (0.90)
Hospitalist	1.23 (0.84)	1.37 (0.80)	1.29 (0.82)
Infectious disease	1.13 (0.70)	1.20 (0.86)	1.17 (0.79)
Total	1.19 (0.88)	1.15 (0.90)	1.17 (0.89)
Proportion reporting burnout scores exceeding threshold indicating burnout			
Emergency medicine	51.4%	45.4%	47.9%
Critical care	53.1%	42.8%	48.3%
Primary care	33.9%	30.2%	31.9%
Hospitalist	28.2%	41.5%	33.8%
Infectious disease	38.4%	35.9%	37.0%
Total	37.5%	33.6%	35.4%

^a^Critical care is an aggregation of the “critical care medicine” and “pulmonary critical care” specialties

^b^Primary care is an aggregation of “internal medicine” and “family medicine” specialties

Conditional mean burnout scores for all respondents increased from 1.11 (CI: 0.86, 1.35) in wave 1 to 1.27 (CI: 1.05, 1.49) in wave 2, an increase of 0.15 (CI: − 0.24, 0.57). In the balanced panel, mean scores increased from 0.91 (CI: 0.52, 1.31) to 1.12 (CI: 0.79, 1.46), an increase of 0.21 (CI: − 0.42, 0.84). Table 3 reports conditional mean PFI-BC scores by physician specialty for both all respondents and for the balanced panel. These are the predicted PFI-BC scores for an average physician of each specialty, accounting for geographic region and the local intensity of the COVID-19 pandemic. In this adjusted analysis, burnout scores increased in wave 2 among all specialties except for emergency medicine, with the largest increases among hospitalists, 0.28 points (CI: − 0.19,0.76) among all respondents and 0.36 (CI: − 0.39,1.11) in the balanced panel, and primary care physicians, 0.21 (CI: − 0.23,0.66) among all respondents and 0.31 (CI: − 0.38,1.00) in the balanced panel. Among hospitalists, the conditional mean increased to 1.38 in wave 2 in the unbalanced panel and to 1.85 in the balanced panel, near or above the 1.4 threshold indicating burnout.

**Table 3:** Estimates of mean PFI burnout score by physician specialty conditional on pandemic confounders for all respondents and balanced panel. Estimated by random effects regression using weighted survey data. Cluster-robust 95% confidence intervals reported in parentheses

	Emergency Medicine	Critical Care^c^	Primary Care^d^	Hospitalist	Infectious Disease
**Panel A. All respondents**^**a**^					
Wave 1	1.47	1.28	1.02	1.10	1.06
	(1.16,1.77)	(1.00,1.55)	(0.74,1.31)	(0.73,1.46)	(0.74,1.38)
Wave 2	1.37	1.44	1.23	1.38	1.29
	(1.10,1.65)	(1.13,1.76)	(0.98,1.49)	(1.02,1.74)	(0.98,1.61)
Change	−0.09	0.16	0.21	0.28	0.23
	(−0.53,0.34)	(−0.25,0.58)	(− 0.23,0.66)	(− 0.19,0.76)	(− 0.20,0.67)
**Panel B. Balanced panel**^**b**^					
Wave 1	1.57	1.11	0.76	1.49	0.69
	(1.08,2.05)	(0.43,1.80)	(0.31,1.22)	(0.69,2.29)	(0.14,1.25)
Wave 2	1.33	1.19	1.07	1.85	0.93
	(0.79,1.88)	(0.49,1.88)	(0.69,1.46)	(1.24,2.46)	(0.40,1.46)
Change	− 0.23	0.07	0.31	0.36	0.24
	(− 0.86,0.39)	(− 0.42,0.57)	(− 0.38,1.00)	(− 0.39,1.11)	(− 0.32,0.79)

^a^All Respondents sample consists of 460 survey responses from 381 physicians. Eighteen responses were dropped due to missing covariate data

^b^Balanced panel consists of 97 physicians whom responded in both waves, for 194 total observations

^c^Critical care is an aggregation of “Critical care medicine” and “Pulmonary critical care” specialties

^d^Primary care is an aggregation of “Internal medicine” and “Family medicine” specialties

Table 4 reports results from conditional quantile regression for both all respondents and the balanced panel. Reassuringly, the conditional median quantile model produces estimates very similar to those produced by random effects regression. Median PFI-BC score increased most among hospitalists, by 0.42 (CI: − 0.10,0.94) among all hospitalist respondents and by 0.77 (CI: − 1.22,2.76) in the hospitalist balanced panel. We explored heterogeneity in the progression of PFI-BC scores in the 25th and 75th quartiles. For all specialties, the PFI-BC burnout scores of physicians in the 25th quartile decreased, while the scores of those in the 75th quartile increased. This explains the somewhat contradictory findings reported in Tables 2 and 3: after adjusting for differences in the intensity of the pandemic, the physicians scoring lowest at wave 1 reported lower scores in wave 2, but the scores of the physicians already feeling burnt out at wave 1 increased at wave 2. The exception to this, however, were hospitalists: burn out either remained constant or increased at every part of the distribution. A fixed effects version of the estimating equation found that conditional mean PFI-BC scores increased, on average, by 0.44 (CI: − 0.44, 1.33) points from wave 1 to wave 2, consistent with point estimates from the random effects and conditional quantile models; fixed effects regression cannot estimate changes by specialty, however.

**Table 4:** Estimates of change in PFI-burnout composite score by physician specialty estimated by conditional quantile regression using weighted survey data. Huber-White robust 95% confidence intervals reported in parentheses

	Emergency Medicine	Critical care^c^	Primary Care^d^	Hospitalist	Infectious Disease
**Panel A. All Respondents**^**a**^					
25th Quantile					
Wave 1	0.86	0.90	0.44	0.65	0.43
Wave 2	0.61	0.54	0.37	0.65	0.57
Change	−0.26	− 0.36	− 0.07	0.00	0.15
	(− 1.07,0.56)	(− 1.02,0.30)	(− 0.40,0.27)	(− 0.68,0.67)	(− 0.35,0.64)
Median Quantile					
Wave 1	1.49	1.42	0.68	1.05	1.07
Wave 2	1.43	1.38	0.98	1.48	1.14
Change	−0.06	−0.04	0.31	0.42	0.07
	(−0.61,0.49)	(−0.76,0.67)	(− 0.34,0.95)	(− 0.10,0.94)	(−0.64,0.78)
75th Quantile					
Wave 1	1.90	1.94	1.73	1.74	1.53
Wave 2	2.01	2.19	1.68	2.18	1.76
Change	0.11	0.25	−0.06	0.43	0.23
	(− 0.74,0.96)	(− 0.57,1.07)	(−1.02,0.91)	(−0.39,1.25)	(− 0.71,1.18)
**Panel B. Balanced panel**^**b**^					
25th Quantile					
Wave 1	1.45	1.06	0.30	0.50	0.44
Wave 2	0.38	0.73	0.28	1.11	0.53
Change	−1.07	−0.33	−0.02	0.61	0.10
	(−2.39,0.24)	(−1.53,0.88)	(−1.27,1.23)	(−0.84,2.07)	(−0.40,0.59)
Median Quantile					
Wave 1	1.52	1.71	0.50	0.99	0.94
Wave 2	1.53	1.76	0.90	1.76	1.20
Change	0.01	0.05	0.40	0.77	0.26
	(−2.09,2.12)	(−1.70,1.80)	(−1.36,2.16)	(−1.22,2.76)	(− 1.20,1.72)
75th Quantile					
Wave 1	2.06	1.90	1.41	2.05	1.65
Wave 2	2.25	2.10	1.52	2.23	1.39
Change	0.19	0.20	0.11	0.18	−0.26
	(−0.93,1.31)	(−0.69,1.08)	(−1.02,1.23)	(−1.21,1.57)	(−1.36,0.84)

^a^ All Respondents sample consists of 460 survey responses from 381 physicians. Eighteen responses were dropped due to missing covariate data

^b^ Balanced panel consists of 97 physicians whom responded in both waves, for 194 total observations

^c^Critical care is an aggregation of “Critical care medicine” and “Pulmonary critical care” specialties

^d^Primary care is an aggregation of “Internal medicine” and “Family medicine” specialties

In the open-ended comments from the first wave survey, the isolation imposed by the pandemic was a prominent theme. Many respondents commented on the visitor restriction impacts for very ill patients*: “The most difficult part of working in the ICU was having patients struggle and die without family members to hold their hands, that was very sad.”*

Some physicians isolated themselves from their own families to protect them: *“While living in the same house I have a separate room and entry/exit door, if I wasn’t in the hospital I was in my room. Eat dinner sitting outside the dining room window in my yard while my wife and kids sat on the other side of the window at the table, so we can have a family meal. Facetime my kids to help them with homework.”*

When asked what changes or modifications would most help them in their work, first wave respondents described a need for more PPE, access to rapid testing for patients, and clear national prevention and treatment guidelines. In the second wave, respondents emphasized increased vaccine availability, clear public health messaging on vaccine effectiveness, and infection prevention measures. In both waves, some respondents across specialties emphasized the importance of the public wearing masks to prevent transmission.

## Discussion

We hypothesized high rates of burnout would worsen over time among frontline physicians during the pandemic. In unadjusted analyses, this was not the case, apart from hospitalists. The overall rate of burnout did not increase in wave 2 responses relative to wave 1, however, mean PFI-BC scores and the proportion scoring in the burnout range did increase for hospitalists, who dealt with increasing caseloads of COVID-19 patients in the winter of 2020, who were often hospitalized for extended periods. In weighted regression analysis, designed to give unbiased estimates adjusting for regional variation in pandemic conditions, rates of burnout increased overall and in all specialties except emergency medicine, among all respondents and in the balanced panel. Emergency medicine physicians, who had the highest rates of burnout among frontline physicians in wave 1, had lower rates of burnout in wave 2, possibly due to institutional or personal adaptations to the pandemic.

In 2019 (before the pandemic), Brady et al. surveyed physicians across all specialties in an AMA Masterfile sample and reported that 44.5% (599) scored 1.4 or greater on the PFI-BC [26]; in the current unadjusted analysis, 40% (184) of the frontline physicians we surveyed during the pandemic scored > 1.4 on the burnout scale in at least one survey wave, suggesting that the relatively high rate of physician burnout pre-pandemic may not have increased at that point. For emergency physicians and critical care physicians, however, who bore much of the initial brunt of the COVID 19 pandemic, mean burnout scores were well above the PFI-BC burnout threshold, and the percentages exceeded the 2019 findings in one or both survey waves.

Our findings are limited by low response rates and potential selection bias. The analysis took several steps to minimize nonresponse bias and ensure representativeness. Substantial imprecision in our estimates is to be expected due to the low response rate and the use of weights to produce representative estimates [27]. Although our findings are not statistically significant at the 5% level, both the random effects and quantile regressions produced similar point estimates consistent with our expectation that burnout scores would be higher in the second wave. Conditional burnout scores increased in the second wave among all respondents and in the balanced panel. Potential selection bias unaccounted for by our analysis may have biased our point estimates toward zero: if physicians most subject to burnout were less likely to respond to a survey, our study would under-estimate true burnout. Lastly, estimates of differences not showing statistical significance should not be interpreted as true zeros without further investigation, as emphasized by the American Statistical Association in 2016 [28, 29]. Issues of identification, plausibility, and external validity – which were addressed rigorously in this analysis - are just as important as *p*-values [30].

Studies from around the world have emphasized the mental health impacts of the pandemic on healthcare worker anxiety, depression, and PTSD [2]. However, the need for a crisis response by physicians may also have had protective effects. Hartzband and Groopman note the pandemic led to an “astounding display of selflessness by healthcare professionals” and restoration of autonomy, competency, and relatedness in a national health crisis may have countered physician burnout trends [31]. In addition, in the first months of the pandemic, public and institutional support for health care workers including physicians, as well as marshalling of internal resources, may have attenuated the extreme demands imposed on frontline physicians by the pandemic, consistent with the JDR theory [16].

Our finding that burnout rates declined somewhat for emergency medicine physicians, while increasing markedly for hospitalists and remaining high for critical care physicians deserves additional research. Critical care physicians care for the most severely ill, and hospitalists care for the greatest volume of inpatients with COVID-19, many of whom have long hospital stays. With the incomplete uptake of the COVID-19 vaccines and the rapid spread of the Delta and then the Omicron variants subsequent to our surveys, all regions of the U.S. again faced increasing rates of infection [32]. It is of note that among all frontline specialties surveyed except emergency medicine, burnout rates increased over the time between survey waves in the conditional analysis, even for those specialties with lower burnout rates. As the pandemic continues despite available vaccines, frontline physician burnout may yet worsen.

## Conclusions

Physician burnout rates were a matter of great concern before the onset of the COVID-19 pandemic. In analyses adjusted for regional variation in pandemic conditions, we found evidence of increasing rates of burnout in the pandemic’s first year, apart from emergency medicine physicians, whose high burnout rates early in the pandemic declined somewhat. Responding to a severe public health crisis may have been protective in some respects, however, as time passes and the pandemic continues, fatigue and frustration among frontline physicians may lead to worsening burnout.

## Data Availability

Fully deidentified datasets used and/or analyzed during the current study are available from the corresponding author on reasonable request.
